# Beta-Lactamase Producing *Escherichia coli* Isolates in Imported and Locally Produced Chicken Meat from Ghana

**DOI:** 10.1371/journal.pone.0139706

**Published:** 2015-10-13

**Authors:** Mette Marie Rasmussen, Japheth A. Opintan, Niels Frimodt-Møller, Bjarne Styrishave

**Affiliations:** 1 Toxicology Laboratory, Analytical Biosciences, Department of Pharmacy, Faculty of Health and Medical Sciences, University of Copenhagen, Copenhagen, Denmark; 2 Department of Microbiology, University of Ghana Medical School, P. O. Box KB 4236, Accra, Ghana; 3 Department of Clinical Microbiology, Rigshospitalet, Blegdamsvej 9, 2100 Copenhagen OE, Copenhagen, Denmark; Amphia Ziekenhuis, NETHERLANDS

## Abstract

The use of antibiotics in food animals is of public health concern, because resistant zoonotic pathogens can be transmitted to humans. Furthermore, global trade with food may rapidly spread multi-resistant pathogens between countries and even continents. The purpose of the study was to investigate whether imported chicken meat and meat from locally reared chicken are potential sources for human exposure to multi resistant *Escherichia coli* isolates. 188 samples from imported and locally produced chicken meat were sampled and analyzed. 153 bacteria isolates were successfully cultured and identified as *E*. *coli* using MALDI-ToF. Of these 109 isolates were from meat whereas the remaining 44 were isolated from the cloaca of locally reared live chickens. Antimicrobial susceptibility test was done on the identified *E*. *coli* isolates. Additionally, beta-lactamases production (ESBL and/or AmpC) were phenotypically confirmed on all isolates showing resistance to cefpodoxime. Beta-lactamase producing (BLP) *E*. *coli* meat isolates were further genotyped. Antimicrobial resistance to four antibiotic markers with highest resistance was detected more frequently in isolates from local chickens compared to imported chickens (tetracycline 88.9% vs. 57.5%, sulphonamide 75.0% vs. 46.6%, ampicillin 69.4% vs. 61.6% and trimethoprim 66.7% vs. 38.4%). Beta-lactamase production was found in 29 *E*. *coli* meat isolates, with 56.9% of them being multiple drug resistant (≥ 3). The predominant phylogroup identified was B1 followed by A and D, with similar distribution among the isolates from meat of locally reared chickens and imported chickens. Beta-lactamase producing genotype *bla*_CTX-M-15_ (50%; 10/20) was the most frequently drug resistant gene detected. More BLP *E*. *coli* isolates were found in imported chicken meat compared to locally reared chickens, demonstrating that these isolates may be spreading through food trade. In conclusion, both imported and locally produced chicken meats are potential sources for human exposure to BLP *E*. *coli*.

## Introduction

The intensive use of antibiotics has increased the frequency of resistance among human pathogens resulting in antimicrobial resistance as an important health problem associated with serious consequences in the treatment of infections of bacterial origin [1]. Resistant bacteria spread across borders via trade and travel, and antimicrobial resistance is therefore considered a global problem [2], although antimicrobial resistance tends to be more significant in low and middle income countries (LMICs) [3].

The development of resistant strains is a natural phenomenon that occurs when microorganisms are exposed to antimicrobial drugs. Use and misuse of antimicrobial drugs accelerates this process and poor infection control practices encourage the spread of antimicrobial resistance [4]. In LMICs such as Ghana, increasing prevalence of multidrug resistant isolates have been reported and very high prevalence of resistance to four antimicrobial agents; tetracycline (82%), ampicillin (76%), chloramphenicol (75%) and cotrimoxazole (73%) was observed among mainly Gram-negative organisms isolated from hospitalized patients [3, 5]. In addition fecal *E*. *coli* isolated from adults in Ghana showed high prevalence of resistance (>80%) to the same four antimicrobial agents [6].

Antimicrobial agents are used in food animals to treat, prevent, and control diseases and in some countries to enhance feed efficiency and weight gain. Some manufactures in Ghana have produced and marketed poultry feed supplements loaded with tylosin (macrolide), chloramphenicol, tetracycline and neomycin (aminoglycoside). These antimicrobials are not approved for use as a feed medication for poultry in other parts of the world [7]. Since antibiotics are easily accessible, farmers in Ghana use and abuse them to compromise farm hygiene [7]. The use of antibiotics in food producing animals is of public health concern because resistant zoonotic pathogens can be transmitted to humans through the consumption or handling of food of animal origin [8]. However, global trade with food may also increase the speed by which pathogens spread across the world. To provide further insight to this important subject, the present study investigated the occurrence of resistant *E*. *coli* in imported chicken meat and in meat from locally reared chickens in Ghana.

## Materials and Methods

### Sample collection

Frozen and defrosted imported broiler chicken thighs as well as locally reared live broiler chickens were included in the study. Imported chicken thighs were purchased in Accra, Ghana, at six different local open markets and in four supermarkets. Purchased meat was immediately put into 6 L sterile plastic bags with ziplock, kept cool in cooler boxes and transported to the laboratory. All local markets were visited twice with a minimum of three weeks between. Locally reared live chickens were purchased at big local open markets in Accra, Ghana. None of these were visited more than once. It was attempted to ensure that none of the chickens came from the same farm and the chickens were collected from different cages. The coordinates for the sampling locations are shown in S1 Table.

All live chickens were purchased as part of the normal diet, in the same way as majority of Ghanaians obtain live chickens for consumption. Live chickens were slaughtered under the supervision of the Veterinary Public Health meat inspector in accordance with the Public Health Act 2012, Act 851 of Ghana and in accordance with the Codex Alimentarius of WHO/FAO and the OIE. A sterile swab stick was used to collect a sample from the cloaca. The chickens were then killed by a hard blow to their head and immediately decapitated. All efforts were made to minimize suffering, and death was instantaneous. To remove feathers, the chickens were then immediately transferred to boiling water after which a sample from the thigh was collected.

### Growth and media

A small portion (approx. 25 g) of fine chopped meat was mixed thoroughly with 225 mL self-prepared peptone water (Oxoid, Denmark) in 500 mL sterile plastic bags with ziplock. The bags were incubated aerobically for 16 ± 2 hours at 37°C. After incubation, 1 μL loop content was streaked onto self-prepared Discovery agar plates (Oxoid, Denmark). A 10 μg cefpodoxime tablet (NEO-SENSITABS) was placed on the most heavily inoculated part of the plate. Since BLPIs have an intrinsic resistance to cefpodoxime, this increases the probability of isolating BLPIs. Hence, plates were scrutinized for the presence of colonies morphologically compatible with E. coli, especially in the cefpodoxime zone, as these isolates were suspicious of being positive for producing betalactamases. The plates were incubated aerobically for 22 ± 2 hours at 37°C. Colonies morphologically compatible with *E*. *coli* were subcultured onto self-prepared MacConkey agar plates (Oxoid, Denmark) and incubated aerobically for 22 ± 2 hours at 37°C. Samples from the cloaca were directly inoculated onto Discovery agar plates and isolated on MacConkey agar plates. Otherwise the procedure was similar to that of the meat samples. All isolated bacteria were kept in agar cryo vials containing 1.8 mL Mueller- Hinton (MH) broth and sent to Denmark for further analysis.

At Hvidovre Hospital, Denmark, all isolates were streaked onto half 5% blood/chrome agar plates (SSI Diagnostica) using a 1 μL loop. The plates were incubated aerobically for 18 ± 2 hours at 37°C. When necessary, colonies were subcultured onto blue plates (SSI Diagnostica) for isolation. To investigate the diversity of *E*. *coli* strains in each meat sample, we initially analysed several isolated *E*. *coli* colonies from 16 samples, 8 from imported chicken and 8 from locally produced chicken using MALDI-ToF MS and phylogenetic typing as described below. In all samples, we only obtained a single type strain. Consequently, it was decided, only to analyse one isolated *E*. *coli* strain per sample.

### E. coli identification

Conclusive identification of all *E*. *coli* isolates were done by the use of MALDI-ToF MS (Bruker BioTyper system version 3.0 (Microflex LT/SH MS)). α-Cyano-4-hydroxy-cinnamic acid (HCCA) was used as matrix. *Escherichia coli* ATCC 25922 was used as a control strain and a score value larger than 2.0 was accepted as a satisfactory accordance between the formed mass spectra and the one in the database. Software program used were FlexiControl and Biotyper real-time classification. Identified isolates were kept in 2 mL freeze broth with 0.9 mL 10% glycerol and stored at -20°C.

### Antimicrobial susceptibility testing

All isolated *E*. *coli* were tested for their antimicrobial susceptibility using disk diffusion test in accordance with the EUCAST guidelines [9]. Strains were grown on Mueller Hinton plates (Becton-Dickinson). *Escherichia coli* ATCC 25922 was used as reference strain. With the exception of sulphonamide (S ≥ 15 mm) and tetracycline (S ≥ 15 mm, R ≤ 11 mm), inhibition zone diameter distributions used were based on breakpoints from EUCAST [10, 11]. A list of all the tested antimicrobials is found in S2 Table.

Phenotypic confirmatory test for detection of ESBL and/or AmpC production was performed on all isolates, which based on results from the antimicrobial susceptibility testing were suspected to be positive, i.e. all organisms with resistance against cefpodoxime. The term ‘resistance’ was applied to organisms that produced an inhibition zone diameter less than 21 mm, or organisms that showed a ‘phantom’ zone. Isolates were tested using disks purchased from MAST (MAST, USA) in accordance to the MAST guideline D68C [12]. The four disks (A-D) used contained: A Cefpodoxime 10 μg, B Cefpodoxime 10 μg + clavulanic acid, C Cefpodoxime 10 μg + cefepime, D Cefpodoxime 10 μg + clavulanic acid + cefepime.

### Classification

Phylogenetic typing was performed on all *E*. *coli* isolates. ESBL as well as AmpC producing *E*. *coli* meat isolates were assigned with a sequence type on the basis of analysis with MLST (multilocus sequence typing) and genotyped.

Bacterial DNA from all isolates were extracted and purified on a MagNA Pure 96 Instrument using Stool Transport and Recovery Buffer (Roche) and a MagNA 96 DNA and Viral NA Small Volume Kit (Version 08, Roche) yielding DNA templates ready for PCR. The PCR was carried out on an Eppendorf Mastercycler gradient instrument. To check whether the PCR successfully generated the desired amplicon(s), agarose gel electrophoresis was carried out using a QIAGEN QIAxcel^®^ instrument together with an alignment marker and a size marker suitable for the respective run. Software program used was QIAxcel ScreenGel version 1.0.2.0. Sequencing was performed by Macrogen’s EZ-seq Service, Holland. Primers used for the sequencing step were identical to those used for PCR amplification. Data analysis, including sequence reviewing and alignment, was performed using CLC DNA Workbench version 6.8.4 (CLC Bio).

#### Phylogentetic typing

All *E*. *coli* isolates were assigned to one of the four phylogenetic groups (A, B1, B2, or D) or designated as non-typeable (NT) based on triplex PCR detection of the two genes *chuA*, *yjaA* and the anonymous DNA fragment TspE4.C2 using QIAGEN Multiplex PCR Kit in accordance with the method described by Clermont et al. [13]. *Escherichia coli* references (ECOR) were used as control strains; ECOR4 (*yjaA* ^-^, *chuA* ^-^, TspE4.C2^-^) as negative control and ECOR20 (*yjaA* ^+^), ECOR48 (*chuA* ^+^), ECOR58 (TspE4.C2^+^), ECOR62 (*yjaA* ^+^, *chuA* ^+^, TspE4.C2^+^) as positive controls. QIAxcel DNA High Resolution Kit for automated analysis of DNA fragments with QX DNA Alignment Marker 15 bp/1kb and a QX DNA Size Marker 50–800 bp were used.

#### MLST

For the detection of the seven *E*. *coli* housekeeping genes (adk, icd, fumC, purA, mdh, recA and gyrB) the multiplex PCR, KAPA 2G Robust HS PCR Kit (Kapa Biosystems), described by Wirth et al. [14] was used. Primers were obtained from Eurogentec, (Seraing, Belgium). QIAxcel DNA Screening Kit QX DNA Alignment Marker 50bp/3kb and a QX DNA Size Marker FX174 Haelll were used. Based on sequencing and data analysis, the allelic profile and sequence types (ST) were obtained via the electronic database at the *E*. *coli* MLST homepage (http://mlst.ucc.ie/mlst/dbs/Ecoli). A cladogram was generated from the MLST data (n = 28; one isolate was excluded since all seven alleles were not readable) using the alignment program ClustalW2 v. 2.1(EMBL-EBI).

#### Genotyping

ESBL positive isolates were screened for *bla*_CTX-M_ genes (1, 2, 8 and 9) using a CTX-M group-specific multiplex PCR, AmpliTaq Gold 360 Master Mix (Applied Biosystems) [15]. An additional PCR specific for the respective CTX-M groups were applied on isolates positive for the *bla*_CTX-M-1_ gene [16]. For the *bla*_CTX-M-1_ positive genes KAPA 2G Robust HS PCR Kit (Kapa Biosystems) was used. ESBL positive isolates were also checked for *bla*_SHV_ and *bla*_TEM_ genes with the use of KAPA 2G Robust HS PCR Kit (Kapa Biosystems) and GeneAmp PCR Reagent Kit with AmpliTaq DNA Polymerase (Applied Biosystems) respectively [17]. QIAxcel DNA Screening Kit QX DNA Alignment Marker 50bp/3kb and a QX DNA Size Marker 50–800 bp were used. Based on sequencing and data analysis, the nucleotide sequences were BLAST (Basic Local Alignment Search Tool) searched using the free NCBI tool BLAST.

AmpC positive isolates were screened for *bla*_MOX_, *bla*_CIT_, *bla*_DHA_, *bla*_ACC_, *bla*_EBC_, *bla*_FOX_ genes using a plasmid-mediated AmpC multiplex PCR, GeneAmp PCR Reagent Kit with AmpliTaq DNA Polymerase (Applied Biosystems) [18]. Primers from Eurogentec, Belgium were utilized. QIAxcel DNA Screening Kit QX DNA Alignment Marker 15bp/1kb and a QX DNA Size Marker 50–800 bp were used.

### Statistics

Fisher's exact test was used to compare frequencies. A *p*-value < 0.05 (two-tailed) was considered to be statistically significant. The data were analyzed using GraphPad Prism software v. 6.0a.

## Results

A total of 188 meat samples were sampled. Of these 56 samples were from locally reared chickens (50 live chickens and 6 from supermarkets) and 132 were imported chicken thighs primarily from the United States (50%) and Brazil (42%). Only four samples (2%) were imported from Holland and the origin of the remaining, approximately 6% was unknown. 153 bacteria isolates were successfully cultured and identified as *E*. *coli*. A total of 109 isolates (71.2%) were from meat whereas the remaining 44 (28.8%) were isolated from feces in cloaca of the locally reared live chickens.

In total, 36 isolates were cultured from the 56 local chickens. From the 132 imported chicken thighs, a total of 73 isolates were cultured. From 29 (65.9%) of the 44 chickens that *E*. *coli* was isolated in feces, *E*. *coli* was also isolated in the meat. Hence for the remaining 15 chickens (34.1%), *E*. *coli* was only isolated from the cloaca.

**Fig 1:**
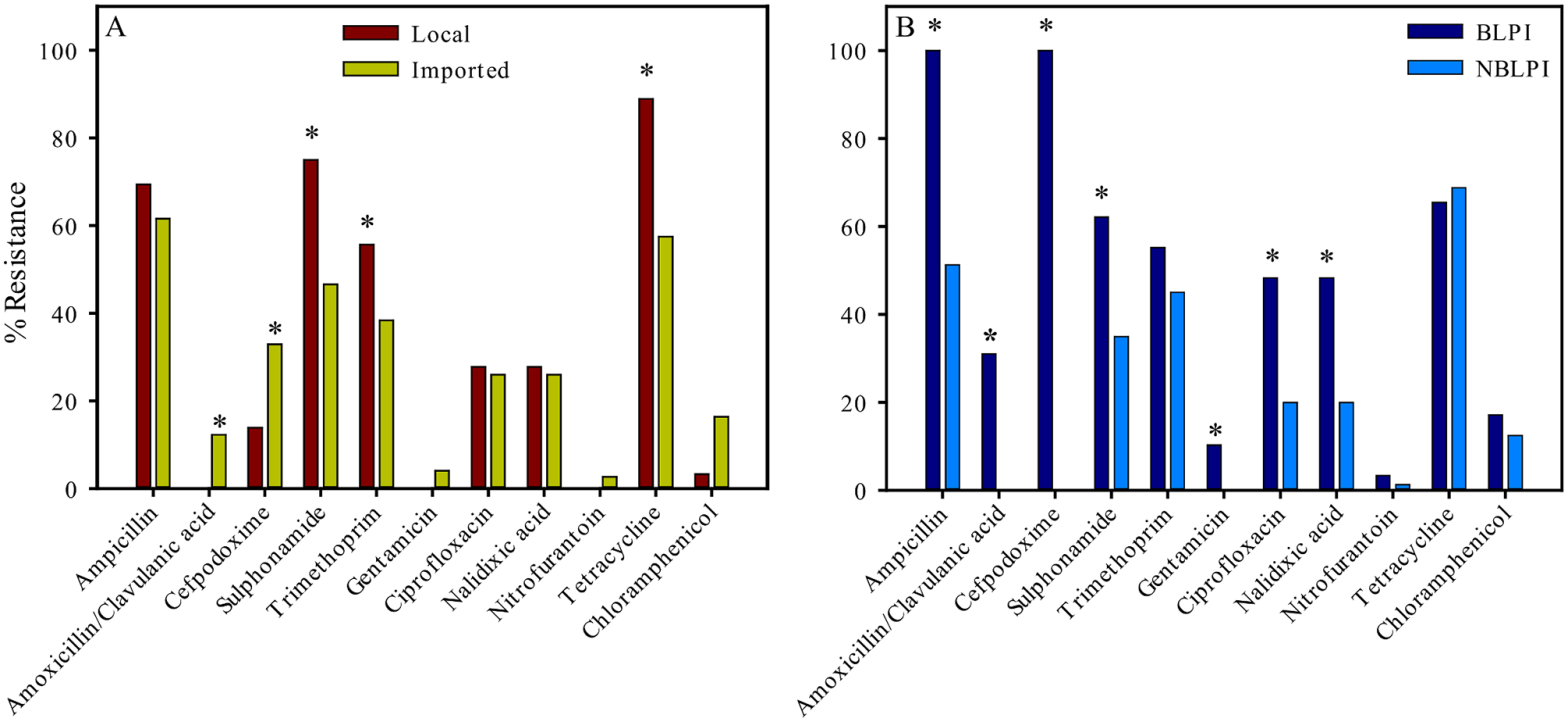
Antimicrobial resistance profile of *E*. *coli* meat isolates. A: Red bars: Strains isolated from local chickens (n = 36); Green bars: strains isolated from imported chickens (n = 73). B: Dark blue bars: Beta-lactamase producing isolates (n = 29). Blue bars: Non beta-lactamase producing isolates (n = 80). * indicates statistical significance (p < 0.05).

The distribution of resistant isolates, sample origin and type (meat or cloaca) are listed in S1 Table. Intermediate resistant isolates were regarded as susceptible. No *E*. *coli* showed resistance to mecillinam, piperacillin/tazobactam, ertapenem and meropenem, and only few isolates showed resistance to gentamicin and nitrofurantoin. Fig 1A shows antimicrobial resistance profiles in *E*. *coli* meat isolates from locally reared chickens (red bars) compared to imported chickens (green bars). Among *E*. *coli* meat isolates from locally reared chickens antimicrobial resistance frequencies were highest for tetracycline (88.9%), followed by sulphonamide (75.0%), ampicillin (69.4%) and trimethoprim (66.7%). As in locally reared chickens, *E*. *coli* meat isolates from imported chickens exhibited highest resistance to the same four antimicrobial agents: ampicillin (61.6%), followed by tetracycline (57.5%), sulphonamide (46.6%) and trimethoprim (38.4%). Isolates from local chickens were significant more resistant to sulphonamide, trimethoprim and tetracycline compared to strains isolated from imported chickens (*p* ≤ 0.0078). Strains from imported chickens were significant more resistant to amoxicillin/clavulanic acid and cefpodoxime (*p* ≤ 0.0398).

Nineteen (19) isolates were found positive for ESBL production only, whereas 9 isolates were positive for AmpC production only. One isolate was positive for the production of both ESBL and AmpC. The distribution of beta-lactamase producing (BLP) isolates among imported chicken meat as well as meat and feces from locally reared chickens is shown in Table 1. Significantly more *E*. *coli* BLP isolates were foun in imported chicken meat compared to locally reared chickens (*p =* 0.0398). Of the 24 *E*. *coli* BLP isolates from imported meat, 13 samples (54.2%) originated from Brazil and the remaining 11 (45.8%) from the US. Thus, no significant difference in origin was established.

**Table 1:** Beta-lactamase producing *E*. *coli* isolates (meat and cloaca).

Beta-lactamase	Meat (n = 109)			Cloaca (n = 44)	Total (%)
	Local (n = 36)	Imported (n = 73)	* *p-*value		
ESBL	4	15	0.2883	1	20 (62.5)
AmpC	0	9	0.0285	2	11 (34.4)
ESBL and AmpC	1	0	0.3303	0	1 (3.1)
**Total**	5	24	0.0398	3	32

*differences in resistance between local and imported meat.

Fig 1B shows antimicrobial resistance profiles of BLP in *E*. *coli* meat isolates compared to non beta-lactamase producing (NBLP) isolates. BLP isolates were 100% resistant to ampicillin and cefpodoxime. In contrast, ampicillin resistance was only detected in 51.3% of all NBLP isolates. The highest antimicrobial resistance was found for tetracycline (65.5%), followed by sulphonamide (62.1%) and trimethoprim (55.2%). For NBLP isolates the highest resistance was observed in tetracycline (68.8%), followed by sulphonamide (53.8%), ampicillin (51.3%) and trimethoprim (45.0%). Except for tetracycline, BLP isolates were more resistant to each antimicrobial tested than NBLP isolates. Since resistance to penicillins and cephalosporins is intrincis to BLP isolates, evidently, resistance was higher for ampicillin, amoxicillin/clavulanic acid and cefpodoxime compared to NBLP isolates. However, this was also significant (*p* < 0.05) for sulphonamide, gentamycin, ciprofloxacin and nalidixic acid. Noteworthy, only a small proportion of all BLP isolates (2/29, 6.9%) were resistant to beta-lactams only. Elevated proportion of co-resistance in BLP *E*. *coli* meat isolates, i.e. resistance to other antimicrobial drug classes associated with resistance to beta-lactam antibiotics, were observed with 22/29 (75.9%), 19/29 (65.5%) and 14/29 (48.3%) to sulphonamide+trimethoprim, tetracyclines or quinolones (ciprofloxacin and nalidixic acid), respectively.

**Fig 2:**
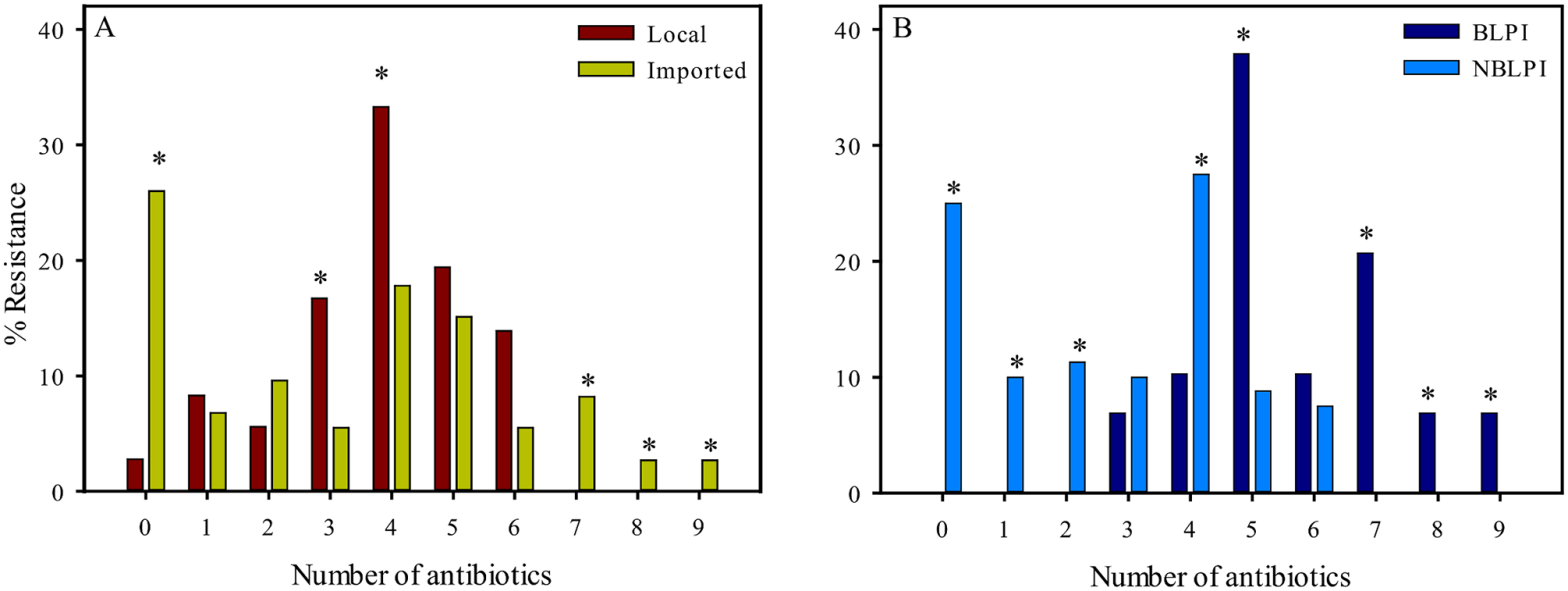
Resistance profile of *E*. *coli* meat isolates relative to the number of antibiotics to which isolates exhibited resistance. A: Red bars: strains isolated from local chickens (n = 36); Green bars: strains isolated from imported chickens (n = 73). B: Dark blue bars: Beta-lactamase producing isolates (n = 29). Blue bars: Non beta-lactamase producing isolates (n = 80). * indicates statistical significance (p < 0.05).

Fig 2A shows *E*. *coli* resistance relative to the number of tested resistant antibiotic markers for isolates from meat of locally reared chickens (red bars, n = 36) and imported chickens (green bars, n = 73). A significantly higher (*p* = 0.0030) proportion of strains isolated from imported meat were fully susceptible with 26.0% vs. only 2.8% of the *E*. *coli* isolates from locally reared chickens. For locally reared chickens some strains were co-resistant to a maximum of 6 antibiotics whereas for imported chickens, strains were co-resistant to up to 9 antibiotic markers. Among the isolates from locally reared chickens, 67% were co-resistant to 4 or more antibiotics whereas for isolates form meat of imported chickens the same was applicable for 2 antibiotics or more. Ten out of 73 *E*. *coli* isolates (13.7%) from imported chicken meat were resistant to 7 or more antibiotics compared to no strains isolated from locally reared chickens.

A comparison of *E*. *coli* BLP isolates (orange bars) and NBLP isolates (green bars) relative to the number of tested resistant antibiotic markers is shown in Fig 2B. Approximately, 38% of the BLP isolates were resistant to five antibiotic markers and two isolates were resistant to nine antibiotics. 25.0% of all NBLP isolates were susceptible to all 15 tested antimicrobials. In general, the NBLP isolates were resistant to a lower number of antibiotics with resistance to a maximum of six antibiotic markers. Approximately 35% of all BLP isolates were resistant to seven or more antibiotics compared to none of the NBLP isolates. *E*. *coli* isolates that were simultaneously resistant to three or more (≥ 3) different antimicrobial drug classes, e.g. beta-lactams, aminoglycosides and quinolones, were defined as being multiple drug resistant. In Table 2, the number of strains exhibiting multi-drug resistance is shown.

**Table 2:** Multiple drug resistant E. coli isolates from locally reared chicken and imported chicken.

**Antimicrobial drug classes**	**Local (n = 36)**	**Imported (n = 73)**	**BLP (n = 29)**	**NBLP (n = 80)**	Total (n = 109)
< 3 (%)	12 (33.3)	35 (47.9)	2 (6.9)	45 (56.3)	**47 (43.1)**
≥ 3 (%)	24 (66.7)	38 (52.1)	27 (93.1)	35 (43.8)	**62 (56.9)**

A similar frequency of *E*. *coli* isolates from meat of imported chickens were found to be resistant to less than three different antimicrobial drug classes (47.9%) compared to those found to be resistant to three or more antimicrobial drug classes (52.1%). Two thirds (66.7%) of the *E*. *coli* isolates from meat of locally reared chickens were found to be multi-drug resistant. However, no statistically significant difference was observed between multi-drug resistant *E*. *coli* isolates from meat of locally reared and imported chickens (*p* = 0.1577). 27 out of 29 (93.1%) BLP isolates were found to be multiple drug resistant compared to 35/80 (43.8%) of the NBLP isolates (*p* < 0.0001). In total, 62 out of the 109 (56.9%) *E*. *coli* isolates from meat were categorized a multi-drug resistant strains.

**Fig 3:**
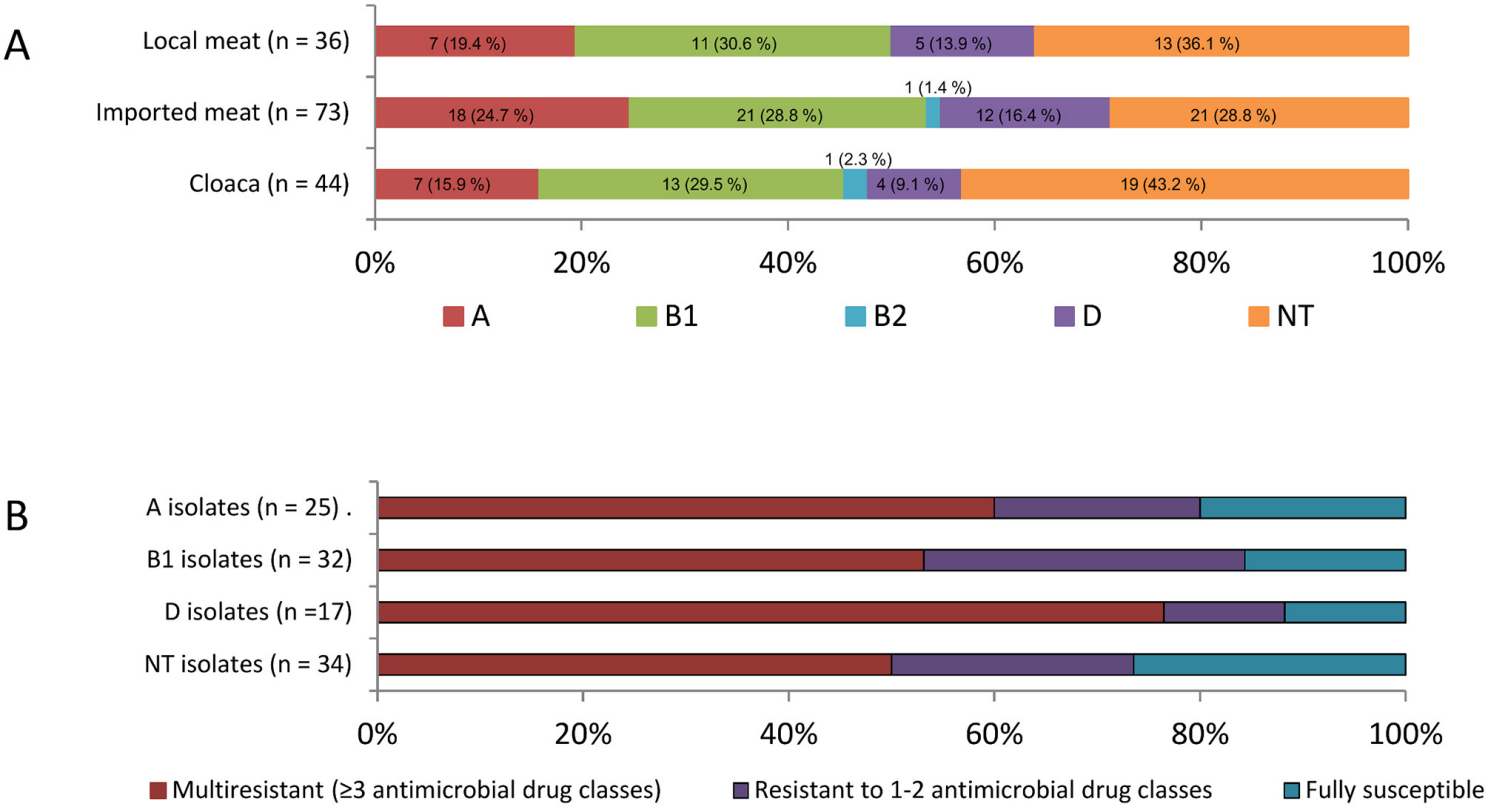
Distribution of phylogroups (A, B1, B2, D) and non-typeables (NT). A: *E*. *coli* isolates originating from meat of locally reared chickens (n = 36), meat from imported chickens (n = 73) and cloaca of locally reared live chickens (n = 44). B: Distribution of antimicrobial frequencies in *E*. *coli* meat isolates (n = 109) into multiresistant isolates (≥3 antimicrobial drug classes), isolates resistant to 1–2 antimicrobial drug classes and fully susceptible isolates divided into phylogroups (A, B1, D) and non-typeable (NT). The B2 phylogroup was not included, as only one isolate, being multiresistant, belonged to this group.

Fig 3 shows the distribution of phylogenetic groups for *E*. *coli* isolates from meat of locally reared chickens, imported chicken thighs and the cloaca of locally reared live chickens. Besides non-typeables, representing the largest group in both sources; cloaca and meat, B1 isolates were the predominant phylogroup, followed by A and D (Fig 3). Only two isolates belonged to phylogroup B2, one each from imported chicken meat and the cloaca of one of the locally reared live chickens. Phylogroups were distributed similarly among isolates from meat of locally reared chickens and imported chickens (*p* ≥ 0.5112). The same was true for the distribution of phylogroups between isolates from meat of locally reared chickens and cloaca (*p* ≥ 0.6472). However, comparison by pairs (*E*. *coli* isolates from meat and feces of the same chicken) revealed that the same phylogroup was only found in 10/29 (34.5%) chickens.

The distribution of phylogroups into multiresistant isolates is also shown in Fig 3. Slightly more phylogroup D isolates were multiresistant compared to the other phylogroup isolates and most susceptible isolates were non-typeable (NT). However, the differences in resistance profiles among typeable and NT isolates was not significantly different (*p* values > 0.0815). Data on phylogroup B2 is not shown as only one isolatebelonged to this phylogroup.

**Fig 4:**
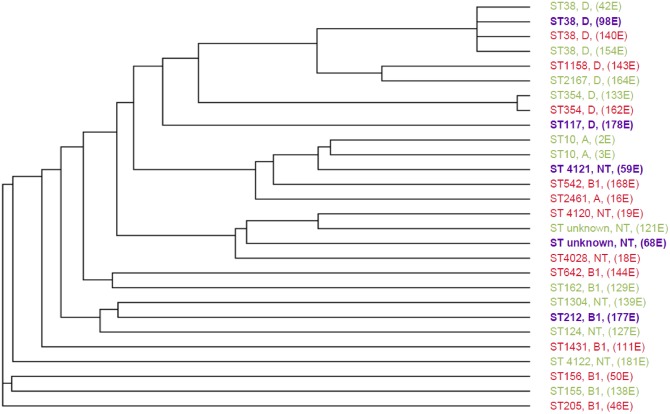
Cladogram showing the relationship of beta-lactamase producing *E*. *coli* isolates typed by MLST (n = 28). STs, phylogroups and sample numbers are shown. Isolates from meat of locally reared chickens (n = 5) are shown in purple. Isolates from meat of chickens imported from the US (n = 11) and Brazil (n = 12) are shown in red and green, respectively. A star (*) indicates ST types that have not been described before.

Table 3 shows MLST results of BLP *E*. *coli* isolates from meat (n = 29). Not all isolates were typeable with the MLST method; for one isolate only readable sequences for six alleles were achievable (denoted NT, non-typeable) and two isolates contained one or two alleles which were not known, and consequently not assigned with an allelic number (denoted as unknown). Finally four isolates had an allelic profile that was unknown in the *E*. *coli* MLST database. These were submitted and given new ST numbers. The remaining 23 out of 29 typed isolates belonged to 19 different STs. Three small clusters were identified: these were ST10, ST38 and ST354, containing two isolates each, except from the ST38 cluster which contained four isolates. MLST cluster analysis showed that isolates from meat of locally reared chickens (purple) did not clustered together (Fig 4).

**Table 3:** Characterization of beta-lactamase producing *E*. *coli* isolates from imported chicken and locally reared chicken. Sample number, origin of chicken, and type of beta-lactamase phenotypically expressed are presented together with phylogroup, ST type, and resistance gene (genotype). Total number of samples = 29. NT (non typeable): ST type was not determined since not all alleles were readable. Unknown: At least one allele was unknown in the database. ST types marked with * had an allelic profile that were unknown in the database and therefore submitted and given a new ST number. Negative: The given isolate was negative for the presence of the resistance genes that were screened for and consequently not determined.

Sample number	Origin	Beta-lactamase	Phylogroup	ST type	Genotype	
					ESBL	AmpC
2E	Brazil	ESBL	A	10	CTX-M-15	-
3E	Brazil	ESBL	A	10	CTX-M/TEM/SHV negative	-
16E	USA	ESBL	A	2461	CTX-M-15	-
18E	USA	ESBL	NT	4028*	CTX-M-15	-
19E	USA	ESBL	NT	4120*	CTX-M/TEM/SHV negative	-
20E	Brazil	ESBL	D	NT	CTX-M group 2 Unknown subtype	-
42E	Brazil	ESBL	D	38	CTX-M-15	-
46E	USA	ESBL	B1	205	CTX-M-15	-
50E	USA	ESBL	B1	156	CTX-M-15	-
59E	Ghana	ESBL	NT	4121*	CTX-M-15	-
68E	Ghana	ESBL	NT	Unknown	CTX-M group 1 Unknown subtype	-
98E	Ghana	ESBL	D	38	CTX-M group 1 Unknown subtype	-
111E	USA	AmpC	B1	1431	-	MOX/CIT/ DHA/ ACC/EBC/FOX negative
121E	Brazil	ESBL	NT	Unknown	CTX-M-15	-
127E	Brazil	AmpC	NT	124	-	CIT
129E	Brazil	ESBL	B1	162	CTX-M-61	-
133E	Brazil	AmpC	D	354	-	CIT
138E	Brazil	ESBL	B1	155	CTX-M-15	-
139E	Brazil	ESBL	NT	1304	CTX-M group 1 Unknown subtype	-
140E	USA	AmpC	D	38	-	CIT
143E	USA	AmpC	D	1158	-	CIT
144E	USA	ESBL	B1	642	CTX-M-15	-
154E	Brazil	ESBL	D	38	CTX-M-1	-
162E	USA	AmpC	D	354	-	CIT
164E	Brazil	AmpC	D	2167	-	CIT
168E	USA	AmpC	B1	542	-	CIT
177E	Ghana	ESBL + AmpC	B1	212	CTX-M/TEM/SHV negative	MOX/CIT/ DHA/ ACC,/EBC/FOX negative
178E	Ghana	ESBL	D	117	CTX-M group 2 Unknown subtype	-
181E	Brazil	AmpC	NT	4122*	-	CIT

Beta-lactamase characterization of all phenotypically expressed *E*. *coli* BLP isolates (n = 29), i.e. both ESBL and/or AmpC positive isolates, was carried out with the use of genotyping. The results revealed that among the 20 ESBL-producing *E*. *coli* isolates 15 produced a CTX-M group 1 enzyme (75.0%) and 2 produced a CTX-M group 2 enzyme (10.0%). Among the 15 CTX-M group 1 isolates, *bla*_CTX-M-15_ with 10/20 isolates (50.0%) was the most commonly detected. *bla*_CTX-M-1_ and *bla*_CTX-M-61_ were only detected in one isolate each. For the remaining three isolates only one readable sequence was achievable, while the CTX-M group 1 subtype of these is unknown. The subtype of the two CTX-M group 2 enzyme-producing isolates was not investigated. AmpC enzymes accounted for resistance in 10 isolates, with the p-AmpC gene *bla*_CIT_, being the most common (8/10, 80.0%). The last two isolates were negative for the presence of all six p-AmpC resistance genes that were screened for.

## Discussion

The present study indicates that global food trade may increase the spread of pathogens such as *E*. *coli* BLP isolates, which is of great concern. In Ghana, imported chicken meat is significantly cheaper than locally produced chicken. An IFAD report from 2006 stated that Ghana’s poultry industry declined steeply in the 1990s after the withdrawal of government support. Moreover, the reduction of tariffs under the influence of the international financial institutions as part of loan conditionality resulted in a significant increase in imported frozen chicken. As these are heavily subsidized, the cost of the imports is artificially low [19]. The consequence may be that the general Ghanaian population far more often purchase imported chicken meat, thereby increasing the likelihood of being exposed to pathogens of foreign origin.

Although it was attempted to ensure that none of the live chickens came from the same farmer by choosing different sellers and consistently only chose broiler chickens that were placed in different cages, it was not possible to exclude the possibility that some chickens came from the same farms. The same was true for imported chicken thighs where it was *de facto* impossible to ensure that chickens were from different wholesalers or batches. However, to eliminate the probability of purchasing imported chicken thighs from the same batch, different markets were visited with a minimum of three weeks in between.

Obeng-Nkrumah [20] demonstrated that 48% of 736 tested health community residents in Ghana harbored fecal BLP *E*. *coli* and *Klebsiella pneumoniae*, predominately, ESBL phenotypes. These ESBL-producing *E*. *coli* phenotypes were identified with the highest frequency in Korle-Gonno 96.4% (134/139), followed by Dodowa 95.7 (91/95) and Tamale 87.1% (108/124). Korle-Gonno, Dodowa and Tamale are in the Greater Accra, Eastern and Northern regions of Ghana, respectively, demonstrating the widespread occurrence of ESBL producing phenotypes in the Ghanaian population. Furthermore, high resistance among *E*. *coli* to the four antimicrobial markers with highest resistance found in the current study (Fig 1), have been reported for several years [21, 22]. Surveillance data show that resistance in *E*. *coli* is consistently highest for antimicrobial agents that have been in use the longest time in human and veterinary medicine [23]. The most resistant phenotypes found in the current study were to tetracycline, sulphonamide and ampicillin, introduced in 1948, 1936 and 1961, respectively [24]. Moreover, the finding of tetracycline resistance as the most common type of resistance observed is not surprising, knowing that tetracycline has been widely used in therapy and as growth promoters in animal food production systems since its approval [25].

The resistance profile observed in the current study was similar to that reported by a recent study in Ghana [26]. In that study, comprising 103 *E*. *coli* isolates from poultry stool specimens, the authors reported a high prevalence of resistance to tetracycline, cotrimoxazole, and ampicillin. Likewise a retrospective study showed that out of 138 *E*. *coli* isolates recovered from chickens between 1970 and 2002, 69% were resistant to tetracycline, 61% to sulphonamide, and 34% to ampicillin [21]. The high occurrence of resistance found in the current study is not surprising knowing that mis- and overuse of antimicrobial drugs in poultry production provide a selective pressure favoring the appearance of resistant strains. In addition, veterinary services are generally considered expensive and farmers might therefore purchase incomplete regiments [27]. Furthermore, the diverse structure in the rules and regulations of medicines makes antibiotics available without prescriptions leading to inappropriate use [28]. All these reasons are known factors in the emergence and spread of antimicrobial resistance.

In the current study, significantly more BLP *E*. *coli* isolates were observed in imported chicken meat than local meat (*p =* 0.0398). Furthermore, the 27% prevalence of BLP *E*. *coli* isolates found in the present study was lower compared to studies from Holland and Spain where prevalences between 66% and 94% have been described [29–31]. This may indicate that the prevalence of BLP *E*. *coli* isolates are higher in industrialised large-scale cicken meat production that in small-scale production.

BLP *E*. *coli* isolates in the current study were 100% resistant to cefpodoxime as this among others is an indicator for detection of beta-lactamase production [32]. Additionally, AmpC isolates showed 100% resistances to amoxicillin/ clavulanic acid as AmpC enzymes in contrast to ESBL is not inhibited by clavulanic acid [33]. Co-resistance to non-beta-lactams was frequently seen in *E*. *coli* BLP isolates, in particularly to sulphonamide+trimethoprim (76%), tetracycline (66%) and quinolones (48%). Such co-resistance in other antimicrobial drug classes associated with resistance to beta-lactam antibiotics is frequently seen in BLP enterobacteriaceae [21, 33]. Emergence of co-resistance may arise as a consequence of long term and intensive use of broad-spectrum antimicrobial agents alone or in combinations.

Tadesse et al. [21] found that 55% of the 138 *E*. *coli* isolates from chickens sample in the US between 1970 and 2002 were multiple drug resistant. This is consistent with the present findings where 56.9% *E*. *coli* isolates from meat were multi drug resistant (Table 2). The fact that 93% of the BLP E. coli isolates were multiple drug resistant was not surprising as most BLP isolates carry additional resistances to drugs such as sulfonamides and other commonly used veterinary drugs [34]. Among the phylogenetic groups (A, B1, B2 and D), isolates from both meat and feces were dominated by B1 isolates, followed by A, D and finally B2 with only two isolates belonging to this group (Fig 3). This phylogenetic distribution profile with significant lower frequency of B2 isolates in both meat and feces of chickens has been reported in previous studies [35–38]. With A and B1 isolates representing ≥ 50% of the isolated *E*. *coli* species from meat, consuming chicken meat may more often expose the intestine among the Ghanaian population to those two phylogroups rather than B2 and D isolates representing ≤ 18%. Although A and B1 isolates may be less successful colonizers of the human intestine compared to B2 isolates [39], A and B1 isolates may predominate the fecal *E*. *coli* transiently from time to time.

It is assumed that the source of virulent human extraintestinal pathogenic *E*. *coli* (ExPEC) clones responsible for diseases, such as urinary tract infections (UTI), originate from the host’s own fecal flora. However, no clear consensus as to how these clones come to inhabit the fecal flora has been established. Based on the identification of avian pathogenic *E*. *coli* (APEC) and human ExPEC, some studies suggests that a potential source of ExPEC clones that colonize human hosts is contaminated poultry meat [35, 36]. If poultry meat serves as a foodborne vehicle for human ExPEC, then potentially zoonotic ExPEC may be present in poultry meat [40].

MLST of the BLP *E*. *coli* meat isolates in the current study demonstrated no major clusters but only one small with four out of 23 typeable isolates assigned to ST 38. Several of the ST types found in the present study have previously been described in chickens. These include ST10, ST117, ST155, ST162, ST1158, ST2167 and ST2461, with ST10 previously characterized in chickens originating from Ghana. All other STs have primarily or exclusively been described in humans with especially ST10 and ST38 being well characterized. Several isolates, ST10, ST156, ST162, ST205, ST212, ST354, ST542 and ST1304, have all previously been detected in humans in Ghana. In addition, this study adds four new STs (ST4028, ST4120, ST4121, ST4122) to the database. The data presented in the MLST analysis indicate that poultry meat may be a potential source of ExPEC clones in the human intestine. However, the diversity of *E*. *coli* BLP isolates (Fig 4) indicates that there was no obvious single source of these strains.

Genotype *bla*_CTX-M-15_ with 10 out of 20 isolates was the most frequent drug resistance gene detected in the current study. Even though geographical differences have been found, the CTX-M-1, CTX-M-14, and CTX-M-15 are within the CTX-M enzymes, by far the most widespread ESBLs not only in humans but also among *E*. *coli* strains from healthy poultry [29, 41–43]. CTX-M-2 was detected in two samples; from one locally reared chicken and one from imported chicken. CTX-M-2 is well known to be the prevalent CTX-M type in clinical isolates from South America and Asia [44], so its isolation from an imported chicken thigh from Brazil is not surprising. Although limited data on the prevalence of resistance gene are available from African countries, it is of high concern that an *E*. *coli* strain with CTX-M-2 was isolated from chicken meat imported to Ghana from Brazil.

The p-AmpC gene *bla*_CIT_ accounted for resistance in 8 out of 10 isolates and was the only AmpC resistance gene detected in the present study. An additional PCR to identify the subtype of the CIT amplicons was not performed, but may be *bla*_CMY-2_ since CMY-2 is by far the most common p-AmpC beta-lactamase enzyme associated with resistance in chickens [38, 41, 45]. P-AmpCs differ from c-AmpCs in being uninducible and are typically associated with broad multidrug resistance [46]. p-AmpC beta-lactamases have been associated with false *in vitro* susceptibility to cephalosporins, a failure that may lead to inappropriate antimicrobial treatment and consequently result in increased mortality. If most clinical laboratories and physicians remain unaware of p-AmpCs clinical importance [47], and if poultry meat is a potential source of ExPEC clones in the human intestine, then the detection of p-AmpC in the present study is alarming.

The present study demonstrates that international trade with food may be a potential source for thespread of ESBLproducing *E*.*coli*. In fecal isolates sampled from 736 healthy community residents in three distant community settings across Ghana, greater than 40%of ESBL-producing *E*. *coli* were positive for *bla*_CTX-M-15_ [20]. Findings from the present study therefore suggest a relationship between chicken meat, drug-resistant *E*. *coli* and the predominant ESBL gene (*bla*_CTX-M-15_) among healthy community residents in Ghana. Urgent steps are needed to decrease ESBL producing *E*. *coli* in local as well as in imported chicken meat.

## Supporting Information

S1 Table Coordinates (latitudes/longitides) for all sampling locations. In total, 8 locations were visited.
(DOCX)

S2 Table Number of resistant isolates to each tested antibiotic. Isolates are divided into origin (cloaca, local meat and imported meat). Percentages are presented in parenthesis.–indicates that no resistance to the given antibiotic was observed.
(DOCX)
