# Supplementary material for: Beta-Lactamase Producing Escherichia coli Isolates in Imported and Locally Produced Chicken Meat from Ghana
Source: PLoS One. 2015 Oct 13;10(10):e0139706. doi: 10.1371/journal.pone.0139706 (PMC4603670; doi:10.1371/journal.pone.0139706)
Supplement: S1 Table — In total, 8 locations were visited. (DOCX) [file pone.0139706.s001.docx]

Coordinates (longitudes/latitudes) for all sampling locations. In total, 8 locations were visited.

| **Sample** | **Location** | **Date** | **Coordinates (latitude/longitude)** |
| --- | --- | --- | --- |
| 1-3 | Kaneshie, Accra,  Greater Accra, Ghana | 2013-02-11 | 5°34'34.02"N  0°14'39.85"V |
| 4-15 | Kaneshie, Accra,  Greater Accra, Ghana | 2013-02-20 | 5°34'34.02"N  0°14'39.85"V |
| 16-28 | Agbogbloshi, Accra,  Greater Accra, Ghana | 2013-02-25 | 5°33'3.83"N  0°12'49.84"V |
| 29-34 | Korle Bu, Accra,  Greater Accra, Ghana | 2013-02-26 | 5°32'14.66"N  0°13'38.63"V |
| 35-44 | Makola Market, Accra, Greater Accra, Ghana | 2013-02-26 | 5°32'51.76"N  0°12'24.71"V |
| 45-56 | Accra New Town,  Greater Accra, Ghana | 2013-02-27 | 5°35'7.16"N  0°12'45.87"V |
| 57-66 | Kaneshie, Accra,  Greater Accra, Ghana | 2013-03-04 | 5°34'34.02"N  0°14'39.85"V |
| 67-76 | Agbogbloshi, Accra,  Greater Accra, Ghana | 2013-03-05 | 5°33'3.83"N  0°12'49.84"V |
| 77-86 | Accra New Town,  Greater Accra, Ghana | 2013-03-11 | 5°35'7.16"N  0°12'45.87"V |
| 87-96 | Mallam Market, Accra, Greater Accra, Ghana | 2013-03-12 | 5°34'19.66"N  0°16'47.52"V |
| 97-106 | Madina,  Greater Accra, Ghana | 2013-03-13 | 5°40'23.26"N  0° 9'58.99"V |
| 107-120 | Kaneshie, Accra,  Greater Accra, Ghana | 2013-03-18 | 5°34'34.02"N  0°14'39.85"V |
| 121-134 | Agbogbloshi, Accra,  Greater Accra, Ghana | 2013-03-19 | 5°33'3.83"N  0°12'49.84"V |
| 135-144 | Makola Market, Accra, Greater Accra, Ghana | 2013-03-20 | 5°32'51.76"N  0°12'24.71"V |
| 145-148 | Korle Bu, Accra,  Greater Accra, Ghana | 2013-03-20 | 0 5°32'14.66"N  0°13'38.63"V |
| 149-162 | Accra New Town,  Greater Accra, Ghana | 2013-03-25 | 5°35'7.16"N  0°12'45.87"V |
| 163-176 | Madina,  Greater Accra, Ghana | 2013-03-26 | 5°40'23.26"N  0° 9'58.99"V |
| 177-188 | Osu, Accra,  Greater Accra, Ghana | 2013-03-27 | 5°33'13.78"N  0°10'30.91"V |

| **Location** | **Coordinates (latitude/longitude)** |
| --- | --- |
| Kaneshie, Accra,  Greater Accra, Ghana | 5°34'34.02"N  0°14'39.85"V |
| Agbogbloshi, Accra,  Greater Accra, Ghana | 5°33'3.83"N  0°12'49.84"V |
| Korle Bu, Accra,  Greater Accra, Ghana | 5°32'14.66"N  0°13'38.63"V |
| Makola Market, Accra,  Greater Accra, Ghana | 5°32'51.76"N  0°12'24.71"V |
| Accra New Town,  Greater Accra, Ghana | 5°35'7.16"N  0°12'45.87"V |
| Mallam Market, Accra,  Greater Accra, Ghana | 5°34'19.66"N  0°16'47.52"V |
| Madina,  Greater Accra, Ghana | 5°40'23.26"N  0° 9'58.99"V |
| Osu, Accra,  Greater Accra, Ghana | 5°33'13.78"N  0°10'30.91"V |
