# Supplementary material for: Beta-Lactamase Producing Escherichia coli Isolates in Imported and Locally Produced Chicken Meat from Ghana
Source: PLoS One. 2015 Oct 13;10(10):e0139706. doi: 10.1371/journal.pone.0139706 (PMC4603670; doi:10.1371/journal.pone.0139706)
Supplement: S2 Table — Isolates are divided into origin (cloaca, local meat and imported meat). Percentages are presented in parenthesis.–indicates that no resistance to the given antibiotic was observed. (DOCX) [file pone.0139706.s002.docx]

Supporting information:

Table 1S:

Number of resistant isolates to each tested antibiotic. Isolates are divided into origin (cloaca, local meat and imported meat). Percentages are presented in parenthesis. – indicates that no resistance to the given antibiotic was observed.

|  |  | Isolates from cloaca,  n = 44 | Isolates from meat, n = 109  ^__________________________________________________________________________________________________________________________________________________________________^ | | |
| --- | --- | --- | --- | --- | --- |
| **Antibiotic** | |  | Local  n = 36 | Imported  n = 73 | **Total** |
| Ampicillin, 10 µg | | 23 (52.5) | 25 (69.4) | 45 (61.6) | 70 (64.2) |
| Mecillinam, 10 µg | | – | – | – | – |
| Piperacillin/Tazobactam 30:6, 36 µg | | – | – | – | – |
| Amoxicillin/clavulanic acid 2:1, 30 µg | | 2 (4.5) | – | 9 (12.3) | 9 (8.3) |
| Cefpodoxime, 10 µ | | 3 (6.8) | 5 (13.9) | 24 (32.9) | 29 (26.6) |
| Ertapenem, 10 µg | | – | – | – | – |
| Meropenem, 10 µg | | – | – | – | – |
| Sulphonamide, 300 µg | | 34 (77.3) | 27 (75.0) | 34 (46.6) | 61 (56.0) |
| Trimethoprim, 5 µg | | 26 (33.8) | 24 (66.7) | 28 (38.4) | 52 (47.7) |
| Gentamicin, 10 µg | | – | – | 3 (4.1) | 3 (2.8) |
| Ciprofloxacin, 5 µg | | 19 (24.7) | 10 (27.8) | 19 (26.0) | 29 (26.6) |
| Nalidixic acid, 30 µg | | 19 (24.7) | 10 (27.8) | 19 (26.0) | 29 (26.6) |
| Nitrofurantoin, 100 µg | | – | – | 2 (2.7) | 2 (1.8) |
| Tetracycline, 30 µg | | 41 (93.2) | 32 (88.9) | 42 (57.5) | 74 (67.9) |
| Chloramphenicol, 30 µg | | 6 (7.8) | 3 (8.3) | 12 (16.4) | 15 (13.8) |
